# Metabolomic analysis of pig spleen reveals African swine fever virus infection increased acylcarnitine levels to facilitate viral replication

**DOI:** 10.1128/jvi.00586-23

**Published:** 2023-08-15

**Authors:** Xing Yang, Xintian Bie, Huanan Liu, Xijuan Shi, Dajun Zhang, DengShuai Zhao, Yu Hao, Jinke Yang, Wenqian Yan, Guohui Chen, Lingling Chen, Zixiang Zhu, Fan Yang, Xusheng Ma, Xiangtao Liu, Haixue Zheng, Keshan Zhang

**Affiliations:** 1 State Key Laboratory for Animal Disease Control and Prevention, College of Veterinary Medicine, Lanzhou University, Lanzhou Veterinary Research Institute, Chinese Academy of Agricultural Sciences, Lanzhou, China; Lerner Research Institute, Cleveland Clinic, Cleveland, Ohio, USA

**Keywords:** African swine fever virus, spleen, acyl-carnitines, fatty acid β-oxidation, metabolomics

## Abstract

African swine fever (ASF) is a devastating disease caused by the African swine fever virus (ASFV) that adversely affects the pig industry. The spleen is the main target organ of ASFV; however, the function of metabolites in the spleen during ASFV infection is yet to be investigated. To define the metabolic changes in the spleen after ASFV infection, untargeted and targeted metabolomics analyses of spleens from ASFV-infected pigs were conducted. Untargeted metabolomics analysis revealed 540 metabolites with significant differential levels. Kyoto Encyclopedia of Genes and Genomes pathway analysis showed that these metabolites were mainly enriched in metabolic pathways, including nucleotide metabolism, purine metabolism, arginine biosynthesis, and neuroactive ligand-receptor interaction. Moreover, 134 of 540 metabolites quantified by targeted metabolomics analysis had differential levels and were enriched in metabolic pathways such as the biosynthesis of cofactors, ABC transporters, and biosynthesis of amino acids. Furthermore, coalition analysis of untargeted and targeted metabolomics data revealed that the levels of acylcarnitines, which are intermediates of fatty acid β-oxidation, were significantly increased in ASFV-infected spleens compared with those in the uninfected spleens. Moreover, inhibiting fatty acid β-oxidation significantly reduced ASFV replication, indicating that fatty acid β-oxidation is essential for this process. To our knowledge, this is the first report presenting the metabolite profiles of ASFV-infected pigs. This study revealed a new mechanism of ASFV-mediated regulation of host metabolism. These findings provide new insights into the pathogenic mechanisms of ASFV, which will benefit the development of target drugs for ASFV replication.

**IMPORTANCE:**

African swine fever virus, the only member of the *Asfarviridae* family, relies on hijacking host metabolism to meet the demand for self-replication. However, the change in host metabolism after African swine fever virus (ASFV) infection remains unknown. Here, we analyzed the metabolic changes in the pig spleen after ASFV infection for the first time. ASFV infection increased the levels of acylcarnitines. Inhibition of the production and metabolism of acylcarnitines inhibited ASFV replication. Acylcarnitines are the vital intermediates of fatty acid β-oxidation. This study highlights the critical role of fatty acid β-oxidation in ASFV infection, which may help identify target drugs to control African swine fever disease.

## INTRODUCTION

African swine fever (ASF) is an acute, febrile, and highly contagious disease caused by the African swine fever virus (ASFV) (1). ASFV is the only member of the *Asfarviridae* family and the only known DNA arbovirus (2, 3). It has a large double-stranded DNA genome with a length of 170–190 kb that contains >150 genes (4). ASFV can infect both domestic and wild boars, including all breeds and ages, with high morbidity and mortality rates; the mortality rate can reach 100% (5, 6). Since 2018, ASF breakouts have been reported in China, the largest producer and consumer of pork worldwide (7, 8). ASF has a devastating impact on the global swine industry and economic trade. Unfortunately, owing to the complex nature of ASFV and limited knowledge of ASFV-host interaction, no safe antiviral drugs for ASF have been developed to date (9). ASFV-infected pigs die immediately and exhibit hemorrhagic necrosis in multiple organs, particularly an ASF-typical lesion, and their spleens are extremely swollen with severe necrosis (10). In the immune system, the spleen is a key tissue that contains many macrophages. ASFV primarily targets cells of the mononuclear phagocytic system, including macrophages (11, 12). Furthermore, transcriptomics and proteomics analyses have revealed that ASFV infection mainly regulates the spleen’s innate immune response and metabolic pathways (13, 14). Thus, the spleen is an appropriate sample for exploring the immune evasion and metabolic mechanisms of ASFV.

High-throughput techniques are a powerful tool for obtaining comprehensive information regarding disease progression. Transcriptomics and proteomics provide data regarding the processes that might occur in the future, whereas metabolomics provides information regarding current processes (15, 16). Metabolomics can reveal the global changes in the levels of small-molecule metabolites caused by viral infection (17, 18). The production of small-molecule metabolites and changes in their levels are the outcomes of biological processes that directly reflect the regulation of viruses (19). Viruses do not possess metabolic capacity and rely on modified host metabolism to replicate (20, 21). Previous studies have revealed that several viruses, such as severe acute respiratory syndrome coronavirus 2 (SARS-CoV-2) (22), Marek’s disease virus (23), infectious spleen and kidney necrosis virus (24), and dengue virus (DENV) (25), regulate host metabolism for promoting their replication. In addition, the hepatitis B virus (HBV) (26), porcine reproductive and respiratory syndrome virus (PRRSV) (27), and classical swine fever virus (CSFV) (28) manipulate metabolites to inhibit the innate immune response. Studies on virus-host interactions have employed metabolomics technology to reveal the molecular mechanisms underlying the virus-induced reprogramming of host metabolism. However, the metabolic changes and functions of the spleen after ASFV infection remain unclear.

This study collected spleen samples from pigs infected with ASFV CN/GS 2018. Mock-infected spleens were used as controls. These spleens were subjected to untargeted and targeted metabolomics analyses. Results revealed significant differential metabolite profiles and metabolic pathways in the spleen after ASFV infection. Importantly, ASFV infection increased the levels of host acylcarnitine, and inhibiting its production and downstream metabolic pathways reduced ASFV replication. These results provide a metabolic basis for further exploring the specific mechanisms underlying ASFV replication.

## RESULTS

### Collection of spleen samples

**Fig 1:**
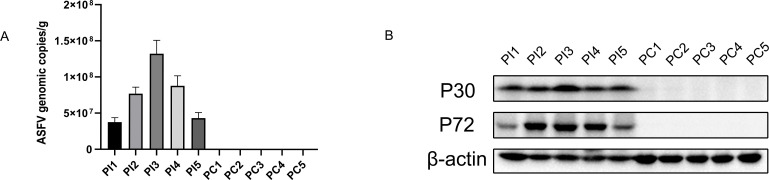
Detection of ASFV replication in pigs. (**A**) Spleen samples were collected from ASFV-infected pigs immediately after euthanasia in the moribund stage; the copy number of the ASFV genome in ASFV-infected spleens was determined using qPCR. (**B**) Viral proteins were analyzed via Western blotting.

The pigs (*n* = 10) were randomly categorized into two groups with five animals each; they were intramuscularly injected with 1 HAD50 of the ASFV CN/CS/2018 (PI group) or an equal volume of phosphate-buffered saline (PBS; PC group). All five pigs infected with ASFV CN/CS/2018 exhibited typical clinical symptoms of ASF, such as depression, anorexia, diarrhea, and high fever, until death (data not shown). Spleen samples were collected from ASFV-infected pigs in articulo mortis, and the protein expression and genome copies of ASFV in these spleens were analyzed via Western blotting and qPCR, respectively. Compared with the PC group, the protein and genome copy levels of ASFV were detected in the five pigs in the PI group, with the number of genome copies reaching >4 × 10^7^ copies/g (Fig. 1A and B).

### Metabolic profiling of spleen samples

**Fig 2:**
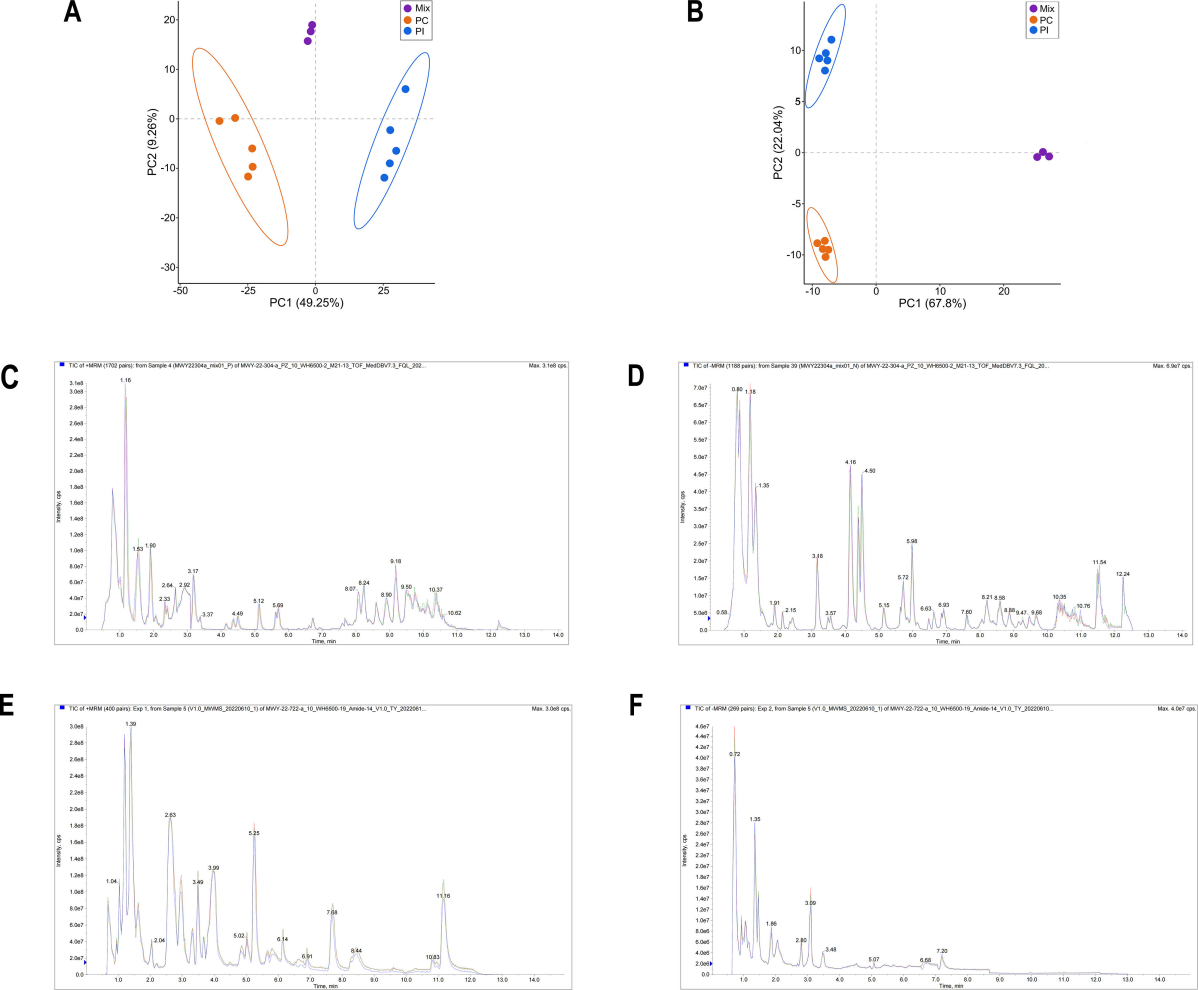
QC analysis of targeted and untargeted metabolomics data. (**A**) PCA charts obtained from untargeted metabolomics analysis; the same amount of PI and PC samples was mixed to obtain a mixed sample. (**B**) PCA charts of targeted metabolomics data; the same amount of PI and PC samples was mixed to obtain a mixed sample. (**C**) TIC overlap diagrams of untargeted metabolomics data in positive ion mode. (**D**) TIC overlap diagrams of untargeted metabolomics data in negative ion mode. (**E**) TIC overlap diagrams of targeted metabolomics data in positive ion mode. (**F**) TIC overlap diagrams of targeted metabolomics data in negative ion mode.

**Fig 3:**
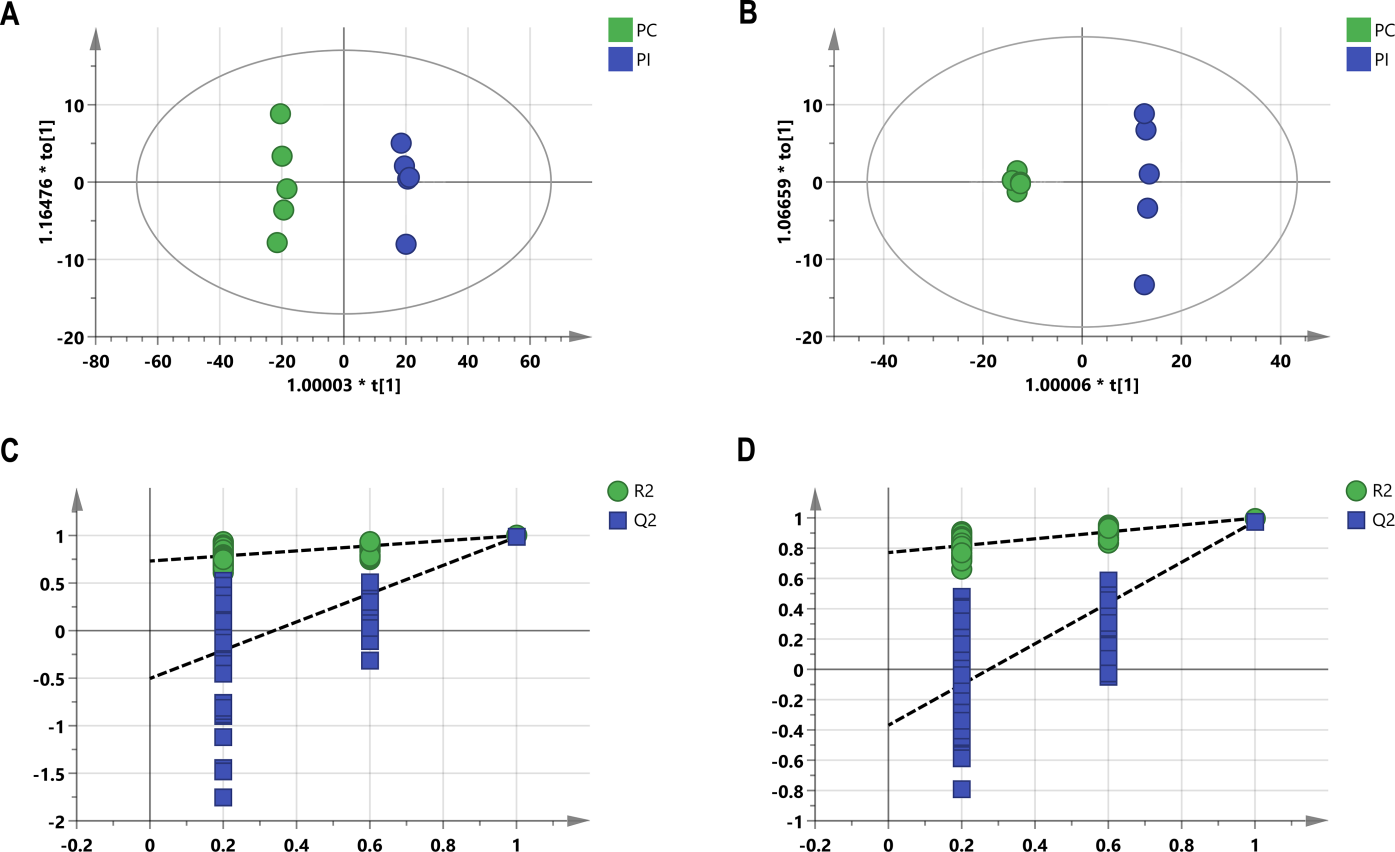
OPLS-DA model obtained from targeted and untargeted metabolomics analyses of spleens from pigs in the PI and PC groups. (**A**) OPLS-DA model of the untargeted metabolomics data. (**B**) OPLS-DA model of the targeted metabolomics data. Permutation tests of the OPLS-DA model for untargeted metabolomics (**C**) and targeted metabolomics analyses (**D**). R2 and Q2 represent the interpretation rate of the model to the matrix and the prediction ability of the model, respectively. R2 and Q2 values closer to 1 indicated a more stable and reliable model.

First, the stability of the analytical method was determined using principal component analysis (PCA) and total ion current (TIC) diagrams. A total of 1,364 metabolites were detected via untargeted metabolomics using hydrophilic and hydrophobic methods. QC samples were clustered together in PCA, indicating the stability of this analytical method (Fig. 2A). TIC diagrams showed that the curves were highly overlapped and that the retention time and peak intensity were consistent (Fig. 2C and D). These results demonstrated that the signals were stable throughout the untargeted metabolomics analysis. As most metabolite classes identified using untargeted metabolomics analysis could also be detected via targeted metabolomics analysis, which quantified 540 metabolites, we selected targeted metabolomics for analyzing the samples to determine absolute metabolite concentrations. Overall, 356 metabolites were quantified by targeted metabolomics analysis. Furthermore, PCA and TIC diagrams indicated the stability of the detection process and the reliability of the analytical method (Fig. 2B, E and F). Moreover, orthogonal partial least squares discriminant analysis (OPLS-DA) was used to analyze nonorthogonal and orthogonal variables and collect more reliable information about the differences in the levels of metabolites between the PI and PC groups. As shown in Fig. 3, a clear separation between the PI and PC groups was observed using both metabolomics analyses (R^2^X = 0.835, Q^2^ = 0.983 and R^2^X = 0.672, Q^2^ = 0.976, respectively) (Fig. 3A and B). Furthermore, the validity of these models was confirmed by 200 permutation tests (Fig. 3C and D). Altogether, these results indicate that the data obtained from both metabolomics analyses are stable and reliable.

### Significant differential metabolite levels and metabolic pathways in the spleen after ASFV infection

**Fig 4:**
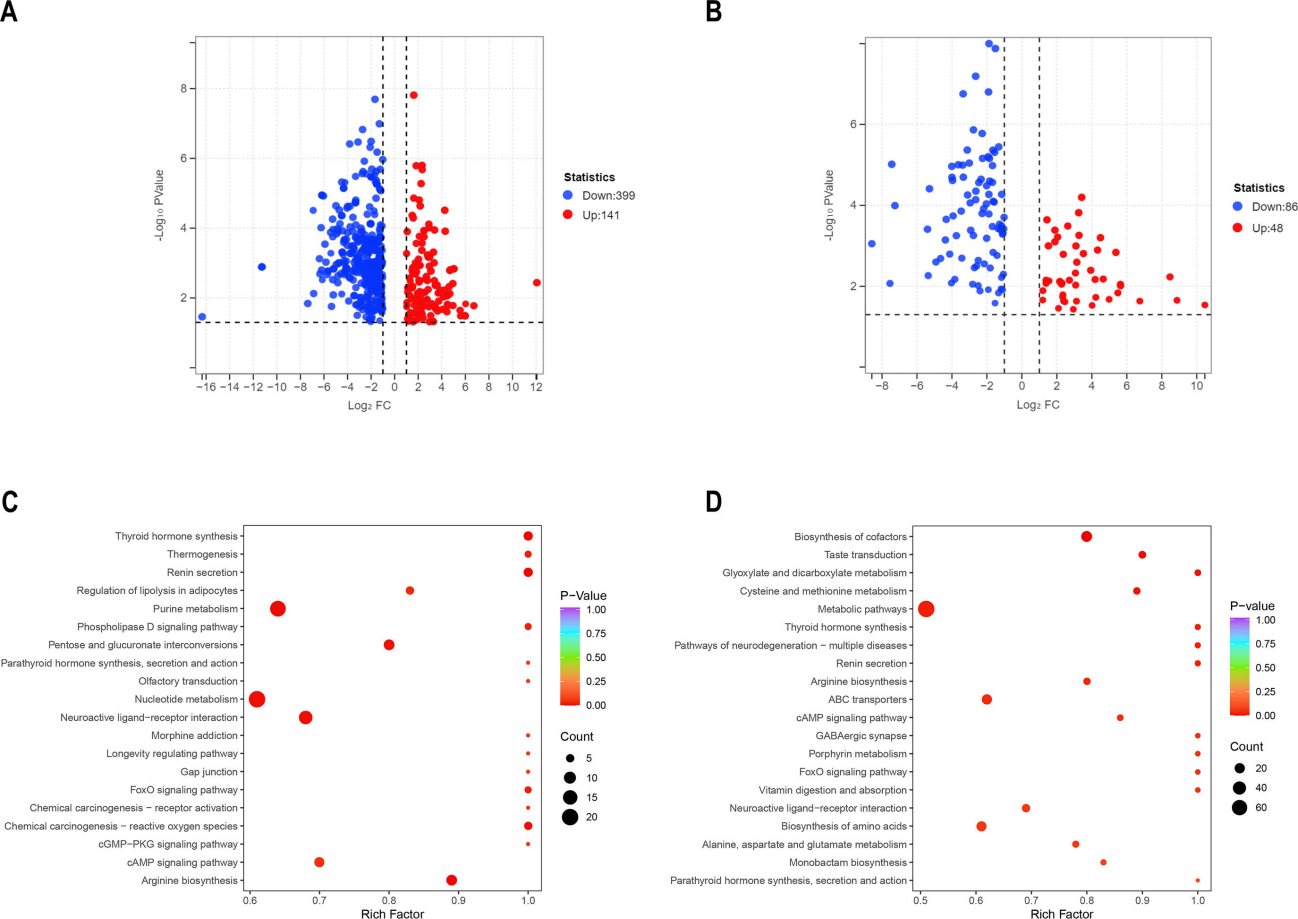
Significant differential metabolite levels and metabolic pathways in pig spleens after ASFV infection. (**A**) Volcano maps of untargeted metabolomics data, with each point representing a significantly altered metabolite. Red indicates upregulation, whereas blue represents downregulation. (**B**) Volcano maps of targeted metabolomics analysis, with each point representing a significantly altered metabolite. Red represents upregulation, whereas blue represents downregulation. Bubble maps of the metabolic pathway enriched by the screened metabolites of the untargeted metabolomics (**C**) and targeted metabolomics (**D**). The X-axis represents the rich factor, and the Y-axis represents the pathway. Each bubble represents a metabolic pathway. The larger the bubble, the more metabolites enriched. The darker the red, the smaller the *P* value, indicating the more significant the enrichment degree; the top 20 metabolic pathways with the highest significance are shown.

Different metabolites were obtained through untargeted and targeted metabolomics analyses to identify the changes in the levels of metabolites after ASFV infection. Untargeted metabolomics analysis revealed that the levels of 540 metabolites were significantly altered after ASFV infection (*P* < 0.05, fold change ≥2 or ≤0.5, and variable important in projection >1); among these, the levels of 399 metabolites were significantly decreased, and those of 141 metabolites were significantly increased (Fig. 4A). Targeted metabolomics analysis showed that the levels of 134 metabolites were significantly altered, with the levels of 86 metabolites being significantly decreased and those of 48 metabolites being significantly increased (Fig. 4B). Subsequently, the Kyoto Encyclopedia of Genes and Genomes (KEGG) Metabolome database was used to identify the corresponding disrupted metabolic pathways based on the differential metabolites after ASFV infection. The top 20 significantly disrupted metabolic pathways revealed by the untargeted and targeted metabolomics analyses were exhibited by bubble plots (Fig. 4C and D). In particular, a KEGG enrichment analysis of untargeted metabolomics data suggested that the metabolites were mainly enriched in nucleotide metabolism, purine metabolism, neuroactive ligand-receptor interaction, arginine biosynthesis, and cAMP signaling pathway. Furthermore, the main enriched metabolic pathways of the targeted metabolomics analysis included the biosynthesis of cofactors, ABC transporters, and biosynthesis of amino acids.

### Levels of acylcarnitines were significantly increased in the spleen of pigs after ASFV infection in both untargeted and targeted metabolomics analyses

**Fig 5:**
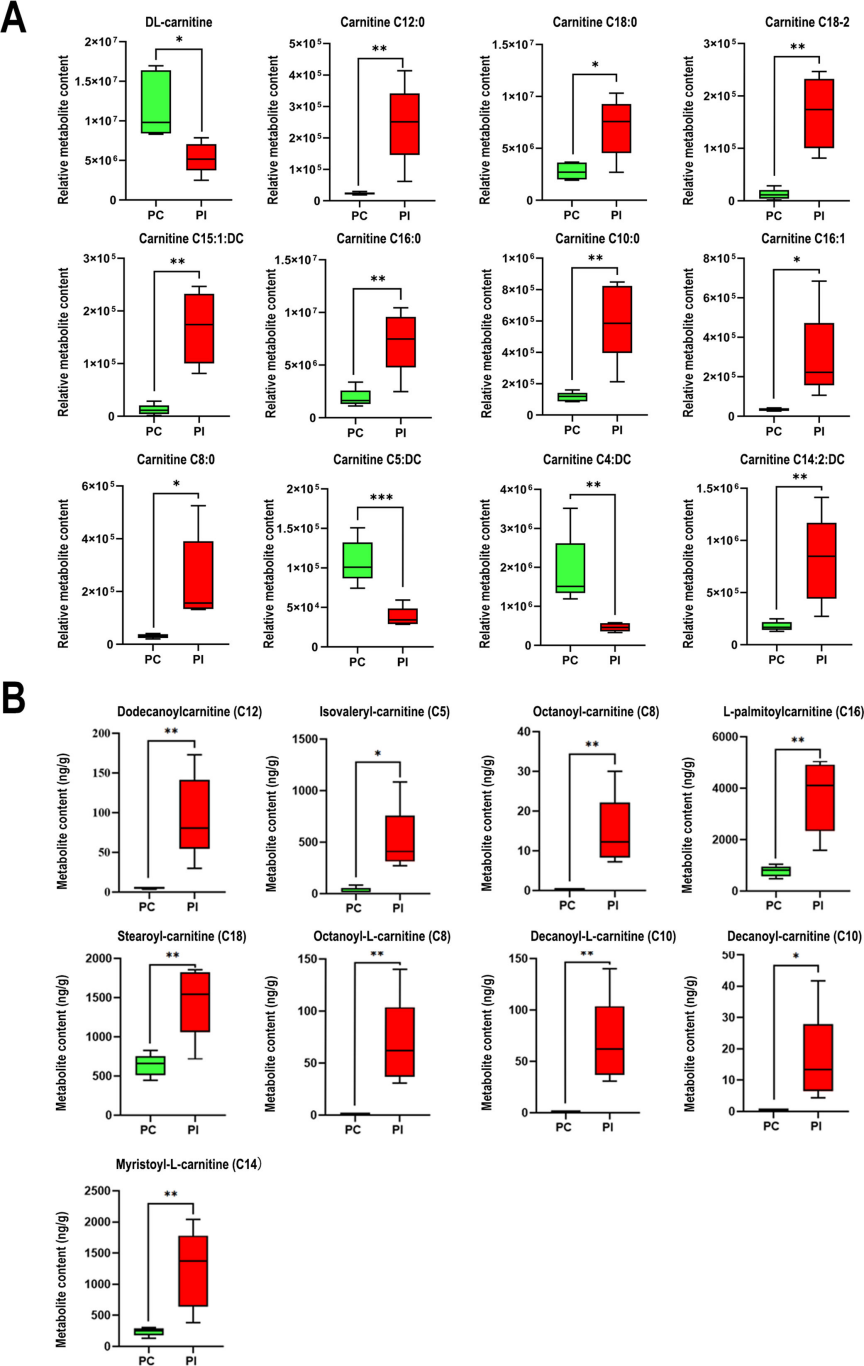
Alteration in the levels of acylcarnitines in the spleens of pigs after ASFV infection. (**A**) Boxplot of significantly altered acylcarnitines in the untargeted metabolomics analysis. Untargeted metabolomics analysis revealed the relative levels of acylcarnitines in each group. (**B**) Boxplot of significantly altered acylcarnitines determined using targeted metabolomics analysis. The levels of acylcarnitines in each spleen sample were determined using a calibration curve of each metabolite in targeted metabolomics. Data are presented as the mean ± SD of five biological replicates in metabolomics.

Both metabolomics analyses revealed that the levels of several metabolites were altered after ASFV infection. To further analyze the metabolic features of the spleen after ASFV infection, we conjointly analyzed the results of the untargeted and targeted metabolomics analyses. A consistent finding in the untargeted and targeted analysis was that acylcarnitine levels were significantly altered after ASFV infection. The untargeted analysis revealed that the levels of 12 acylcarnitines were significantly altered, i.e., carnitines C12:0, C18:0, C18:2, C15:1:DC, C16:0, C10:0, C16:1, C8:0, and C14:2:DC were significantly upregulated, and only DL-carnitines, carnitine C5:DC, and carnitine C4:Dc were significantly downregulated (Fig. 5A). The targeted analysis showed that the levels of eight acylcarnitines were significantly altered (all increased), including dodecanoylcarnitine (C12:0), isovalerylcarnitine (L-carnitine isoC5:0), octanoyl-carnitine (L-carnitine chloride C8:0), L-palmitoylcarnitine (C16:0), myristoyl-L-carnitine (L-carnitine chloride C14:0), stearoyl-carnitine (C18:0), octanoyl-L-carnitine (C8:0), decanoyl-L-carnitine (C10:0), and decanoyl-carnitine (L-carnitine chloride C10:0) (Fig. 5B). Furthermore, the levels of L-palmitoylcarnitine (C16:0) and dodecanoylcarnitine (C12:0) were increased in both metabolomics analyses, and L-palmitoylcarnitine (C16:0) showed the highest content in the spleen after ASFV infection. These results indicated that ASFV infection increased the levels of acylcarnitines, which may help ASFV replication.

### Inhibition of acylcarnitine relative metabolism hindered ASFV replication

**Fig 6:**
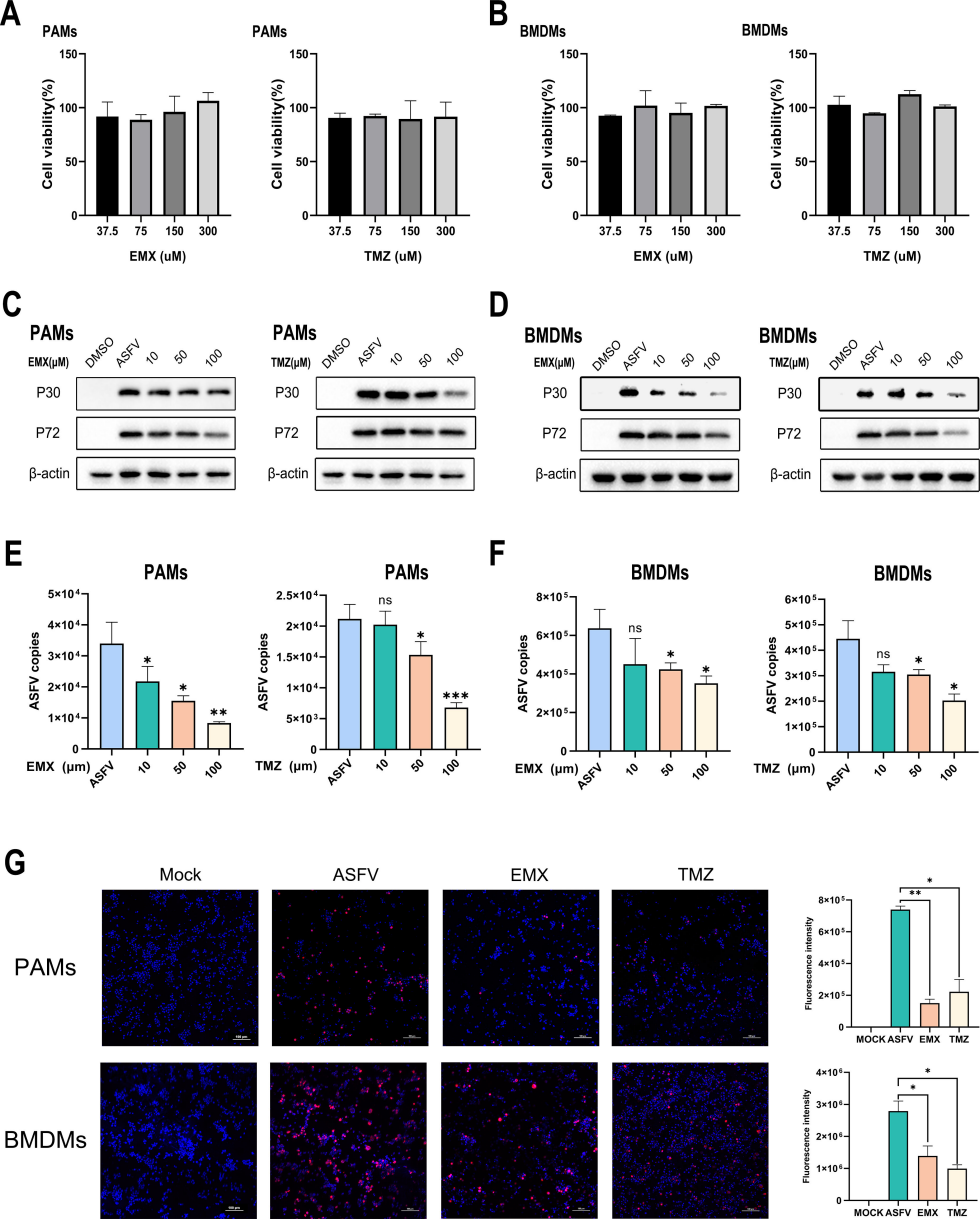
Inhibition of the metabolism of acylcarnitines suppressed ASFV replication. (**A**) The cell cytotoxicity of EMX and TMZ on PAMs was quantified using Cell counting kit-8 (CCK-8) assay, respectively. (**B**) The cell cytotoxicity of EMX and TMZ on BMDMs was quantified using CCK-8 assay, respectively. (**C** and **D**) Effects of EMX and TMZ on viral protein expression. PAMs (**C**) or BMDMs (**D**) were infected with equal amounts of ASFV (MOI = 0.1) and treated with increasing concentrations of EMX or TMZ (10, 50, and 100 µM), respectively. The same volume of DMSO (dimethyl sulfoxide) was added as a mock infection. At 24 hpi, the expression of viral proteins was analyzed using Western blotting via anti-P30 and anti-P72 antibodies. (**E** and **F**) The change of the ASFV genome copies on PAMs and BMDMs after treatment with increasing concentrations of EMX or TMZ was detected using qPCR. (**G**) PAMs and BMDMs were cultured in 20-mm culture dishes, infected with ASFV (MOI = 0.1), and treated with 100 µM EMX and TMZ, respectively. After 24 hpi, these cells were treated with anti-P30 and fluorescence-labeled antibodies; images were acquired under a confocal microscope, and the red fluorescence intensity of each picture was measured through image J.

Acylcarnitines are intermediates of fatty acid β-oxidation that shuttle fatty acyl-CoA from the cytoplasm into the mitochondria for subsequent energy (ATP) production (29). Although the levels of several acylcarnitines were increased after ASFV infection, their role in ASFV replication remains unclear. Thus, to explore the effects of acylcarnitines on ASFV replication, porcine alveolar macrophages (PAMs) and bone marrow-derived macrophages (BMDMs) were treated with etomoxir (EMX) and trimetazidine (TMZ), respectively. EMX is a small molecule that irreversibly inhibits the activity of carnitine palmitoyltransferase 1a, the mitochondrial enzyme responsible for forming acylcarnitines (14). TMZ is a competitive inhibitor of 3-ketoacyl coenzyme A thiolase, a key enzyme in oxidation, located downstream in acylcarnitine metabolism (30). Treatment with different concentrations of EMX and TMZ did not cause significant cytotoxicity in PAMs and BMDMs (Fig. 6A and B). PAMs and BMDMs were cultured in 6-well plates infected with ASFV (MOI = 0.1) and treated for 24 h with increasing concentrations of EMX or TMZ, respectively. The expression of P30 and P72, which were analyzed using Western blotting, indicated that EMX and TMZ inhibited ASFV replication in a dose-dependent manner (Fig. 6C and D). qPCR yielded the same results (Fig. 6E and F). In addition, IFA validated these results (Fig. 6G). These data confirmed that acylcarnitines and fatty acid β-oxidation are essential for ASFV replication.

## DISCUSSION

Recent studies have helped improve the understanding of metabolic changes and their roles during viral infection. Metabolomics techniques have become an increasingly powerful tool for determining metabolite changes and further analyzing the interaction between viruses and hosts. For instance, HBV infection regulates innate immunity by activating glycolysis, inhibiting retinoic acid-inducible gene I-induced interferon production (26). SARS-CoV-2 infection hijacks the folate and carbon metabolism of the host for viral replication (31); moreover, amino acid and fatty acid metabolism are important for this process (32). Furthermore, Zika virus infection reprograms the placental lipidome to support viral replication and provokes inflammation of the human placenta (33). In the current study, we first analyzed the metabolite profiles of the spleen from ASFV-infected pigs using targeted and untargeted metabolomics analyses. This study provides additional information on ASFV-host interactions *in vivo* through metabolomics approaches, which may help understand its pathogenesis and the development of targeted drugs.

Viruses are obligate intracellular parasitic microorganisms. To complete their life cycle, viruses must reprogram the host environment for their benefit. Metabolites are low molecular weight (<1,500 Da) intermediates or products of chemical reactions catalyzed by various enzymes in living systems and end products of cellular processes (34). Thus, compared with transcriptomics and proteomics, metabolomics can more realistically elucidate the underlying mechanisms regulated by viruses (15). Here, we have provided data regarding the changes in metabolite levels and metabolic pathways in pig spleens after ASFV infection. OPLS-DA was used to indicate significant changes in the levels of metabolites after ASFV infection. Subsequently, we analyzed the significant metabolites and associated KEGG enrichment pathways in ASFV-infected spleens. The top 20 KEGG enrichment pathways identified by the untargeted and targeted metabolomics analyses revealed significant changes in amino acid metabolism (biosynthesis of amino acids, such as arginine, cysteine, and methionine), nucleotide metabolism, and lipid metabolism (phospholipase D signaling pathway and regulation of lipolysis in adipocytes). Previous studies have shown that L-arginine can promote ASFV replication (35) and that ASFV infection increases aspartate and glutamate levels to promote its replication (36). Nucleotide metabolism has been changed in ASFV-infected PAMs (36), consistent with our results. Moreover, lipids metabolism has changed after ASFV infection. Nucleotides and lipids are essential substances for the life cycles of various viruses, such as Zika, IAV, and SARS-CoV2 (37 - 40). The changed lipids and nucleotides may play an important role in the life cycle of ASFV. In summary, amino acid, nucleotide, and lipid metabolism changes can partly reflect how ASFV manipulates the host metabolism to promote self-replication. Nevertheless, the detailed mechanism underlying how ASFV regulates the host metabolism to promote self-replication warrants further research.

Acylcarnitines are the intermediates of fatty acid metabolism. Fatty acid metabolism includes synthesis and catabolism, and fatty acid β-oxidation is the key step in fatty acid catabolism. During fatty acid β-oxidation, fatty acids are activated (FA-CoAs) in the cytoplasm and esterified with carnitine to produce acylcarnitines under the catalytic action of carnitine acyltransferase; they are then shuttled to the mitochondrial matrix for ATP production (41 - 43). Viruses that regulate fatty acid β-oxidation to promote their replication have been widely reported. Vaccinia virus upregulates β-oxidation in green monkey BSC40 cells to produce more ATP for protein synthesis and viral replication (44). Fatty acid β-oxidation is also increasingly required during DENV infection (45). In contrast, SARS-CoV2, HCV, JEV, and IAV downregulate β-oxidation, which provides free fatty acids in the cytoplasm for viral replication (46 - 49). However, the changes in acylcarnitine levels during the upregulation/downregulation of β-oxidation may exhibit a different trend. Here, acylcarnitines were significantly changed in both the untargeted and targeted metabolomics analyses, with almost all of them being increased. The accumulation of acylcarnitines has two potential explanations. One possibility is that β-oxidation is inhibited or blocked during ASFV infection, as demonstrated in cells exposed to hypoxic conditions (50, 51). Another possibility is that ASFV upregulates β-oxidation, resulting in the entry of several FA-CoAs into the mitochondria, which exceeds the capacity of β-oxidation. In the human skeletal muscle, insulin resistance exhibits the same phenomenon (52). The ATP and acetyl-CoA produced by β-oxidation are used to meet the need for viral replication. Zika virus and ZENV infection increase the levels of acylcarnitines in Aag2.TET cells, and exogenous addition acylcarnitines promote viral replication, thus supporting this hypothesis (29). Here, our results showed that ASFV replication was significantly reduced by inhibiting the production of acylcarnitines and downstream β-oxidation, indicating that ASFV can upregulate acylcarnitines to promote self-replication through β-oxidation. Nevertheless, the strategy used by ASFV to upregulate acylcarnitines remains unclear and will be one of our future research directions.

In conclusion, our study demonstrated the disruption of metabolism and differential metabolite levels in the spleen of ASFV-infected pigs. These findings may help improve our understanding of the pathogenic mechanisms of ASFV. Moreover, this study confirmed that the intermediate molecules of fatty acid β-oxidation acylcarnitines accumulated in ASFV-infected spleens and that inhibiting fatty acid β-oxidation significantly reduced ASFV replication. This study identified a new strategy of ASFV hijacking host metabolism for self-replication, which may guide the design of targeted drugs for treating ASF.

## MATERIALS AND METHODS

### Cells, viruses, antibodies, and reagents

Porcine alveolar macrophages and bone marrow-derived macrophages were prepared as previously described (53, 54). Both cell types were cultured in Roswell Park Memorial Institute 1640 medium containing 10% porcine serum at 37°C under 5% CO_2_. The ASFV CN/GS/2018 strain was provided by the Lanzhou Veterinary Research Institute (LVRI), Chinese Academy of Agricultural Sciences (CAAS). Viral titration was performed using the hemadsorption assay, with the results presented as 50% hemadsorbing dose (HAD50) per milliliter. Anti-P72 (B646L) polyclonal and anti-P30 (CP204L) monoclonal antibodies were prepared and provided by our laboratory. Horseradish peroxidase-conjugated goat anti-mouse IgG (H1L; SA00001-1) was purchased from Proteintech (Chicago, IL, USA). Etomoxir and trimetazidine were purchased from Selleck (Houston, TX, USA). Cell Counting Kit-8 reagent was purchased from APExBIO (Houston, TX, USA).

### Animal infection and sample collection

Animal experiments were performed in enhanced biosafety level 3 facilities at LVRI of CAAS. According to the Animal Ethics Procedures and Guidelines of the People’s Republic of China, these experiments were conducted strictly with good animal practice. This study was approved by the Animal Ethics Committee of LVRI of CAAS.

Landrace pigs (age, approximately 75 d; weight, 25–30 kg; and *n* = 10), which were free of porcine reproductive and respiratory syndromevirus (PRRSV), pseudorabies virus (PRV), porcine epidemic diarrhea virus (PEDV), and porcine circovirus type 2 (PCV2), were obtained from a high-health farm. Pigs were randomly categorized into two groups (five pigs infected with 1 HAD50 of ASFV CN/GS/2018 and five pigs injected with an equal volume of PBS). Spleen samples were collected from ASFV-infected pigs (PI group) immediately after euthanasia in the moribund stage, and the spleen samples of mock-infected pigs (PC groups) were collected simultaneously. Then, they were frozen via immersion in liquid nitrogen and stored at −80°C.

### Metabolite extraction

First, 20 mg of each sample was collected and homogenized at 30 Hz for 20 s. Then, 400 µL of 70% methanol in water (internal standard extractant) was added, and the samples were shaken for 5 min at 1,500 rpm. Subsequently, the samples were centrifuged for 10 min at 12,000 rpm and 4°C after incubation in an ice bath for 15 min, and the supernatant was collected in a new Eppendorf tube and incubated at −20°C for 30 min. Finally, the samples were centrifuged for 3 min at 12,000 rpm and 4°C, and the supernatant was collected for further analysis.

### Untargeted metabolomics analysis

The collected supernatant (2 µL) was injected into an HSS T3 column (100 × 2.1 mm, 1.8 µm; Waters, Milford, MA, USA) at 40°C using Agilent 1290 Infinity UPLC system (ExionLC AD). The mobile phase consisted of solutions A (water containing 0.1% formic acid) and B (acetonitrile containing 0.1% formic acid). The gradient elution procedure was as follows: 95:5 A/B at 0 min, 10:90 A/B at 10.0 min, 10:90 A/B at 11.0 min, 95:5 A/B at 11.1 min, and 95:5 A/B at 14.0 min. The flow rate was 0.4 mL/min.

Spectrograms were acquired using AB Triple TOF 6600 (AB SCIEX, Danaher, Washington, DC, USA) mass spectrometer on an information-dependent basis. In each cycle, 12 precursor ions with an intensity of >100 were selected for fragmentation at a collision energy of 30 V (12 MS/MS with a production accumulation time of 50 ms each). Electrospray ionization (ESI) was performed in positive and negative ion modes. The operation parameters were as follows: ion source gas 1, 50 psi; ion source gas 2, 50 psi; curtain gas, 25 psi; source temperature, 500°C; and ion spray voltage floating, 5,500 or −4,500 V in positive or negative mode, respectively.

### Targeted metabolomics analysis

The sample extracts were analyzed using LC-ESIMS/MS system (UPLC, ExionLC AD, QTRAP 6500 + System). The ESI positive and negative ion modes were used for MS detection. A 6500 QTRAP mass spectrometer (AB SCIEX) was used to obtain spectrograms. The operation parameters of ESI were as follows: an ion source, ESI^+/−^; source temperature, 550°C; IS, 5,500 V (positive) or −4,500 V (negative); and curtain gas, 35 psi.

### Western blotting

The protein samples were analyzed using 10% sodium dodecyl sulfate-polyacrylamide gel electrophoresis and transferred to NC membranes (EMD Millipore, Billerica, MA, USA). Then, the membranes were blocked with 5% skim milk at room temperature for 1.5 h. After washing thrice with Tris-buffered saline containing 0.1% Tween, the membranes were incubated overnight with the indicated antibodies at 4°C. The membranes were again washed thrice and incubated with secondary antibodies at room temperature for 2 h. Finally, antigen-antibody complexes were observed using an electrochemiluminescence solution, and images were acquired using Odyssey infrared imaging system.

### Real-time quantitative polymerase chain reaction

ASFV genomic DNA was extracted from cells, homogenates, and sera using QIAamp DNA Mini Kits (Qiagen, Germany). Subsequently, the copy number of the ASFV genome was detected as previously described (55). qPCR was performed using Pro Taq HS Premix Probe qPCR kit (Accurate Biology, China) via QuantStudio5 system (Applied Biosystems, USA). The TaqMan probe and P72 primers used for qPCR were as follows: P72-F: 5′-GATACCACAAGATCAGCCGT-3′, P72-R: 5′-CTGCTCATGTATCAATCTTATCGA-3′; TaqMan: 5′-CCACGGGAGGAATACCAACCCAGTG-3′.

### Indirect immunofluorescence assay

PAMs and BMDMs were incubated in dedicated cell confocal imaging dishes. After ASFV infection and treatment with the indicated inhibitor for 24 h, the cells were fixed with 4% paraformaldehyde for 30 min, permeabilized with 0.2% Triton X-100 for 10 min, and blocked with 5% BSA for 1 h. Next, the cells were incubated with anti-P30 antibodies for 10 h at 4°C, followed by Alexa Fluor 568 anti-mouse IgG for 2 h and stained with 4-methyl-6-phenylindole for 10 min. The samples were detected using Leica SP2 confocal system (Leica Microsystems).

### Cell viability assay

Cells were seeded in a 96-well plate (10^5^ cells/well) and treated with different concentrations of inhibitors for 24 h. The cytotoxic effect of the drugs on cells was evaluated using Cell counting kit-8 assay, following the manufacturer’s protocol.

### Data processing and analysis

The raw data’s peak alignment, calibration, retention time, and peak area were extracted for metabolomics analysis using XCMS software (https://xcmsonline.scripps.edu/index.php). To assess the changes in the levels of small-molecule metabolites after ASFV infection, unsupervised PCA and orthogonal partial least squares discriminant analysis were performed using SIMCA-P 14.1 software package. The quality of OPLS-DA models was described using R^2^X and Q^2^ values. In addition, the identified metabolites were annotated using the Kyoto Encyclopedia of Genes and Genomes to identify the metabolic pathways that were altered during ASFV infection.

All data from three independent tests performed *in vitro* were analyzed using GraphPad Prism v.8 (San Diego, CA, USA) and presented as the mean ± standard deviation. *P < 0.05, **P < 0.01, and ***P < 0.001 were considered statistically significant.
